# When are clinical trials registered? An analysis of prospective versus retrospective registration

**DOI:** 10.1186/s13063-016-1310-8

**Published:** 2016-04-15

**Authors:** Stephanie L. Harriman, Jigisha Patel

**Affiliations:** BioMed Central, 236 Gray’s Inn Road, London, WC1X 8HB, UK

## Abstract

**Background:**

Due to problems of publication bias and selective reporting, the ICMJE requires prospective registration of all clinical trials with an appropriate registry before the first participant is enrolled. Previous research has shown that not all clinical trials are registered at this time (prospectively). This study investigated the extent and timing of trial registration. The aims were to determine 1) the proportion of clinical trials that were registered prospectively or retrospectively and 2) when retrospective registration took place in relation to submission to the journal in which they were published.

**Methods:**

All clinical trials published in the *BMC* series in 2013 were identified. All articles that met the study’s inclusion criteria were categorised into one of three categories: 1) prospectively registered, 2) retrospectively registered before submission to the journal in which they were published or 3) retrospectively registered after submission to the journal in which they were published.

**Results:**

One hundred and eight eligible studies were identified. Of these, 33 (31 %) reported studies that were registered prospectively, 72 reported studies that were registered retrospectively (67 %) and three articles (3 %) did not include a trial registration number. Of the 72 studies that were registered retrospectively, 66 (92 %) were registered before the article was submitted to the journal and six (8 %) were registered after the article was submitted to the journal.

**Conclusions:**

Ten years after the ICMJE requirements for prospective registration of clinical trials this study found that the majority of included clinical trials were registered retrospectively but before submission to a journal for publication. This highlights the need for organisations other than journals, such as research institutions and grant giving bodies, to be more involved in enforcing prospective trial registration.

## Background

Many studies are never published [1], in particular, ‘negative’ studies, which are less likely to be published than those reporting a positive outcome [1–4]. For those clinical trials that are published, there is evidence of selective reporting; outcomes are incompletely reported, statistically significant outcomes are more likely to be reported, and primary outcomes are changed [5]. Collectively, these problems distort the evidence on which clinical decisions are based. Clinical trial registration aims to address these problems through the creation of a public record of the existence of all clinical trials. Since July 2005, member journals of the International Committee of Medical Journal Editors (ICMJE) have required registration of clinical trials to counter problems with selective reporting and non-publication of negative results [6].

Clinical trials are defined by the World Health Organisation (WHO) as “*any research study that prospectively assigns human participants or groups of humans to one or more health-related interventions to evaluate the effects on health outcomes.*” [7]. Registration should be with an appropriate registry and should take place before enrolment of the first participant [8].

Despite these requirements, there is evidence that unregistered clinical trials are published [9]. We also know from our past experience as journal editors that journals receive submissions of manuscripts describing clinical trials that have not been registered before the first participant is enrolled. The journals of the *BMC* series consider retrospectively registered clinical trials, as do other journals [10]. Few studies have investigated the extent of retrospective registration of clinical trials and at what point clinical trials are registered.

The aim of this study was to determine:

1. the proportion of clinical trials published in the *BMC* series journals during 2013 that were registered prospectively and the proportion that were registered retrospectively
2. for those studies that were registered retrospectively, whether registration took place before or after submission of the results to the journal in which they were published.

## Methods

The setting for this study was the *BMC* series, which is a group of open access, peer-reviewed journals spanning all areas of biology and medicine. It includes a series of subject-specific journals, two highly selective journals publishing articles of special importance or broad interest and one journal publishing all sound science across all fields of biology and medicine.

We identified all clinical trials published in all journals of the *BMC* series between 1 January and 31 December 2013. To do this, a Structured Query Language (SQL) query was written to extract all relevant articles from an internal reporting database. The query identified all articles that were published in any of the *BMC* series journals in 2013 where the title or abstract contained the word ‘trial’. All identified articles were then screened to identify those that met the following inclusion criteria:

- publication in a journal of the *BMC* series in 2013
- publication as a ‘Research Article’ article
- reporting outcomes of a clinical trial, according to the WHO definition of a clinical trial at the time of publication of the article.

Studies were excluded if they met the following exclusion criteria:

- patient enrolment began before July 2005
- articles reporting only secondary analysis of results from previously reported clinical trial
- articles where the data reported had already been published
- studies that did not meet the WHO definition of a clinical trial
- pilot studies for which no health outcomes were reported and which solely reported on feasibility of a larger study.

All articles meeting the above inclusion criteria were searched for the following information:

- the name of the journal in which the article was published
- the presence of a trial registration number
- the study design (for example, randomised controlled trial (RCT), interventional non-randomised)
- the date of submission of the manuscript to the journal in which it was published
- the country of the affiliation given for the first author
- whether any of the authors declared a financial competing interest.

Where there was a trial registration number available, the registry record was searched for the following information:

- the name of the registry in which the trial was registered
- the date on which the trial was registered with the trial registry
- the date of enrolment of the first participant (as specified in the trial registry record).

Once all eligible articles had been identified and the above data extracted, included articles were categorised as follows:

1. **Prospectively registered**: defined as those where the date of registration recorded in the trial registry record was earlier than the date of enrolment as recorded in the trial registry record.
2. **Retrospectively registered before submission to journal**: defined as those where the date of registration recorded in the trial registry record was later than the date of enrolment as recorded in the trial registration record, but before the date of submission of the manuscript to the journal.
3. **Retrospectively registered after submission to journal**: defined as those where the date of registration recorded in the trial registry record was later than the date of submission of the manuscript to the journal.

For all studies that were retrospectively registered, but registered prior to submission to the journal in which they were published, the length of time between the start date of the study (patient enrolment) and the date of registration was recorded.

Some trial registries include both the date of submission/application for trial registration and the date that the trial registration number was granted in the trial registry record; however, both dates are not available in all registries. For those registries where both dates are available, date of registration was used. For registries where only date of application/submission is available (clinicaltrials.gov), this date was taken as the date of registration. For those registries where only date of registration is available (ChiCTR, DRKS, NTR and UMIN-CTR), this date was used.

Terminology for the date of enrolment of the first participant differs between trial registries; for example, in clinicaltrials.gov it is given as ‘study start date’ and in ISRCTN as ‘anticipated start date’. Where only one date was given (clinicaltrials.gov and ISRCTN), this was considered to be the date of enrolment of the first participant. Where only the month and year were available (clinicaltrials.gov), the date of enrolment was taken to be the last day of the month. Where a trial registry report gave both an anticipated and actual date of enrolment (ANZCTR), the actual date was considered to be the date of enrolment.

The results are presented as absolute numbers with percentages and as medians with interquartile ranges where appropriate.

## Results

**Fig. 1:**
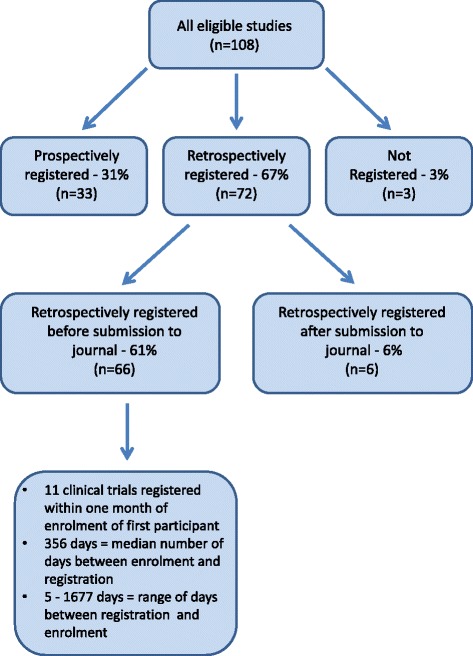
Number (and % of total included studies) of identified clinical trials registered at different stages

One hundred and eight articles met the study’s inclusion criteria and were included in this study (see Fig. 1). Characteristics of the included studies are shown in Table 1. The articles were published across 21 different journals within the *BMC* series, all of which were medical journals. The clinical trials they reported were registered in eight different trial registries. The most common study design reported was RCT (83). Thirteen articles reported other interventional studies that did not involve randomisation and 12 reported pilot RCTs (where a health outcome was assessed in addition to feasibility of the intervention for a larger study). Based on country of affiliation of the first author, included trials were from 30 countries across six continents. The largest group of studies were from Europe (47 %). Financial competing interests were declared in 16 (15 %) of the included articles. See Table 2.

**Table 1:** Characteristics of included studies: number and percentage of included articles/studies by journal in which the article was published, trial registry in which the trial was registered, study design, continent of the first authors’ affiliation, and presence or absence of a declared financial competing interest

Study characteristics	All studies (% of total 108)
Journal in which published	
- *BMC Anaesthesiology*	1 (1)
- *BMC Cancer*	2 (2)
- *BMC Complementary and Alternative Medicine*	10 (9)
- *BMC Cardiovascular Disorders*	2 (2)
- *BMC Dermatology*	1 (1)
- *BMC Emergency Medicine*	3 (3)
- *BMC Endocrine Disorders*	1 (1)
- *BMC Family Practice*	11 (10)
- *BMC Gastroenterology*	8 (7)
- *BMC Geriatrics*	9 (8)
- *BMC Health Services Research*	5 (5)
- *BMC Infectious Diseases*	6 (6)
- *BMC International Health and Human Rights*	1 (1)
- *BMC Medicine*	7 (6)
- *BMC Musculoskeletal Disorders*	2 (2)
- *BMC Nephrology*	9 (8)
- *BMC Pediatrics*	8 (7)
- *BMC Pregnancy and Childbirth*	4 (4)
- *BMC Psychiatry*	7 (6)
- *BMC Public Health*	9 (8)
- *BMC Pulmonary Medicine*	2 (2)
Trial registry	
- ANZCTR	11 (10)
- CHiCTR	3 (3)
- Clinicaltrials.gov	63 (58)
- DRKS	3 (3)
- IRCT	2 (2)
- ISRCTN	15 (14)
- NTR	4 (4)
- UMIN-CTR	4 (4)
Study design	
- RCT	83 (77)
- Pilot RCT	12 (11)
- Interventional, non-randomised	13 (12)
Continent of affiliation (first author)	
- Africa	2 (2)
- Asia	24 (22)
- Australia	11 (10)
- Europe	51 (47)
- North America	14 (13)
- South America	6 (6)
Financial competing interests (CI) of any author	
- Financial CI declared	16 (15)
- No financial CI declared	92 (85)

**Table 2:** Distribution of 108 included articles by study characteristics (study design, continent of first author’s affiliation, and presence or absence of a declared financial competing interest) and registration status (prospective, retrospective and unregistered)

	Prospectively registered studies (*n* = 33)	Retrospectively registered studies (*n* = 72)	Unregistered studies (*n* = 3)
Study design			
- RCT	26	56	1
- Pilot RCT	4	7	1
- Interventional, non-randomised	3	9	1
Continent of affiliation (first author)			
- Africa	1	1	0
- Asia	6	17	1
- Australia	3	8	0
- Europe	17	33	1
- North America	5	8	1
- South America	1	5	0
Financial competing interests (CI) of any authors			
- Financial CI declared	8	8	0
- No financial CI declared	25	64	3

Of the 108 articles, 33 (31 %) reported studies that were registered prospectively, 72 reported studies that were registered retrospectively (67 %) and three articles (3 %) did not include a trial registration number. Of the 72 studies that were registered retrospectively, 66 (92 %) were registered before the article was submitted to the journal and six (8 %) were registered after the article was submitted to the journal.

For studies that were registered retrospectively, but before submission to the journal in which they were published, registration took place between five and 1677 days after enrolment of the first participant. The median number of days between enrolment of the first participant and registration was 356 (IQR 109.3–868). Eleven studies were registered within one month (30 days) of enrolment of the first participant. We also looked at whether there were differences in time of registration within different fields. Median and IQR were calculated for the four journals that published the highest number of clinical trials that were registered retrospectively before submission to the journal (*BMC Complementary and Alternative Medicine, BMC Family Practice, BMC Pediatrics and BMC Public Health*) (see Table 3).

**Table 3:** Median number of days between first participant enrolment and registration for retrospectively registered studies in the four journals publishing the largest number of clinical trials

Journal	Median days to registration	Interquartile range
*BMC Complementary and Alternative Medicine*	491	448 to 1172
*BMC Family Practice*	418.5	139.5 to 676.25
*BMC Pediatrics*	745	243 to 1273
*BMC Public Health*	321.5	148.5 to 1239.25

There were three articles that were categorised as not including a TRN. Two of these did not include a TRN in the published article and no record of the clinical trial could be found by searching the International Clinical Trials Registry Platform search portal. The other article did contain a TRN for the registry EudraCTR; however, we were unable to locate the record for this clinical trial using the TRN given. The corresponding author for each of these articles was contacted by email to ask whether the study had been registered. One confirmed that the study was not registered, one did not reply, and the unobtainable EudraCTR number was confirmed but it was still not possible to locate the record. This was therefore categorised as not registered.

## Discussion

The majority of clinical trials reported in the articles included in this study were registered retrospectively, but before submission to the journal in which they were published. Only a very small number (*n* = 3) did not report a TRN. This compares favourably with a previous study of a sample of RCTs which found that the TRN was reported in the published article for 55 % of RCTs indexed in MEDLINE and 60 % of RCTs registered in NTR [11]. It is also lower than a study of RCTs published in the ten highest ranked surgical journals by impact factor which found that 35 % of included clinical trials were not registered [12].

There are several possible reasons why a clinical trial may not be registered prospectively. Some researchers may not be aware of trial registration and the need to register their clinical trial. Researchers may be aware of trial registration, but not realise that their study meets the definition of a clinical trial, particularly those that are not RCTs. It is also possible that researchers may deliberately choose not to register to avoid publishing ‘negative’ results or to enable them to change primary outcomes or selectively report outcomes. Low support among trialists for trial registration has previously been observed, with a 2007 survey finding that only 21 % of respondents had registered all on-going trials since 2005 and only 47 % stated they would register future clinical trials [13].

Clinical trials that were registered retrospectively were registered at different times between first participant enrolment and submission to the journal in which they were published. This would appear to reflect a lack of importance applied to trial registration. Although it is done eventually, it appears to be seen as merely an administrative exercise that can be done at any time. Registration around the time of participant enrolment may be due to different members of the research team conducting different elements of the process of setting up the clinical trial, or due to the large number of different tasks trialists must complete at this stage. Registration nearer the time of submission may be due to an earlier submission to another journal from which the manuscript was rejected, or the researchers reading the journal’s ‘Instructions for Authors’. Retrospective registration after submission is likely to be in response to the Editor requesting that the authors register the study.

ICMJE policies require registration of clinical trials before enrolment of the first participant, and editorial policies of member journals reflect this [6]. Despite this, journals receive submissions of articles reporting clinical trials that have not been registered prospectively. Editors must decide whether to consider such submissions.

There are ethical arguments for and against consideration of clinical trials that were not registered prospectively. Retrospective registration negates many of the benefits of trial registration such as preventing non-publication of so called ‘negative’ studies, selective reporting, or changing of primary outcomes. Considering retrospectively registered clinical trials may also undermine attempts to increase prospective registration by removing some of the incentive for researchers to register prospectively.

Conversely, clinical trials involve human participants who have taken part in medical research, giving their time and exposing themselves to risk on the understanding that the research will be published and potentially benefit future patients. By refusing to publish retrospectively registered clinical trials, journals would be causing non-publication of research involving humans and also contributing to publication bias. For this reason, there have also been calls for the retrospective registration of all trials that were not registered in the past [14–16].

If journals do consider clinical trials that were not registered prospectively, they should define the conditions under which such trials would be considered. For example, one option would be to only publish retrospectively registered clinical trials if researchers were genuinely unaware of the requirement for registration. While editors can ask authors for an explanation as to why their trial was not prospectively registered, they would not be able to ascertain with certainty that the researcher truly did not know that their trial required registration, potentially making this option unfeasible. In addition, not requiring retrospective registration of all unregistered studies means that if the manuscript is rejected, there is no publicly available record of that clinical trials’ existence, which may lead to non-publication elsewhere.

Another option could be for journals to include date of registration of the trial, a ‘retrospectively registered’ flag, or an explanation from the authors for why the trial was not registered prospectively. This would make the registration status of a publication more transparent, allowing readers to judge the research accordingly. The *BMC* series currently asks authors to indicate the date the trial was registered when presenting the trial registration number in the abstract.

There is little research into timing of trial registration, and this study provides important information highlighting the extent of retrospective registration of published clinical trials. Our results differ from those of Huser and Cimino, who found that 60 % of clinical trials were registered before participant enrolment. There are a number of possible reasons for this. There are differences in the journals included; their study included only the five ICMJE founding journals with 2011 Impact Factors of ≥10.0 [9]. These journals differ in a number of ways from the *BMC* series, including Impact Factor and threshold. Our methodology also differs to that used by Huser and Cimino. We applied strict criteria for classifying a study as retrospectively registered. Those registered the day after enrolment of the first participant were counted as retrospective. This is based on similar criteria used by trial registries that add a ‘retrospectively registered’ flag to trial registry records [personal communication with ISRCTN]. Husser and Cimino applied more generous criteria, allowing a 60-day grace period, to account for the 21-day grace period allowed by US law [17] and data quality assurance processes [9].

Compared to our results, Hardt et al. also found a lower percentage of retrospectively registered studies when looking at registration of RCTs published in surgical journals [18]. Again, these included journals may have differed to the *BMC* series in that the study by Hardt et al. looked only at the ten highest ranked (by Impact Factor) surgical journals. Our study also used the WHO definition of a clinical trial, which is broader than just RCTs. In another study involving surgical journals, this time looking at RCTs published in the ten surgical journals with the highest Impact Factors, Killeen et al. found that 21 % were registered after completion of the study [12].

Our study also shows a greater percentage of retrospectively registered clinical trials in contrast to other studies of retrospective registration of clinical trials. For example, Viergever and Ghersi [19, 20] found that around half were retrospectively registered. This difference may be due to differences in study design, as these studies looked at a sample of trial registry records [19, 20], while our study looked at published articles. The clinical trials registered in the trial registry may differ from those clinical trials published in the *BMC* series.

### Limitations

Our study has a number of limitations. It is limited by the variation in presentation and quality of information as well as the amount of required information between different trial registries. For example, there is a lack of consistency between registries in the date given for registration. While some registries publish both date of submission of the application and date registration was granted, some registries only include date of submission to the registry or date of granting registration. There is also a lack of consistency between terminology regarding study start dates, and accuracy of this date; for example, registries may report ‘study start date’, ‘anticipated start date’, or ‘actual start date’. The quality clinical trial registry records have previously been identified as inconsistent [19, 20].

Although it has never actively encouraged it, the *BMC* series has always been willing to consider retrospectively registered studies. This may mean its journals receive a greater number of submissions and consequently publications reporting retrospectively registered studies compared to other journals that either do not publish retrospectively registered studies or have only recently changed to consider them. This may be one reason for the high number of retrospectively registered studies identified in our study compared with Huser and Cimino’s [9].

Another limitation is that we do not know whether manuscripts had been previously rejected from other journals before submission to the *BMC* series journal in which they were published. During an earlier submission to another journal, authors may have been asked to retrospectively register their trial. We were not able to determine how far this occurred in our sample of articles.

It is possible that the *BMC* series’ reputation for considering retrospectively registered clinical trials discouraged researchers from registering prospectively. Although journals do play a role in promoting prospective trial registration via policies that incentivise prospective registration, they are not well placed in the process of scientific research to ensure that prospective registration takes place. Except in the case of study protocols, editors do not encounter clinical trials until long after they are completed, at which point it is too late. Furthermore, it is unlikely that any single journal editor or publisher can change the behaviour of researchers who do not view clinical trial registration as an important part of the research and publication process. As long as authors have somewhere to publish their findings, journal editors and publishers remain relatively weak. Other stakeholders, earlier in the process, need to be involved in setting out internationally agreed requirements, similar to those for the ethical conduct of human research, which journal editors and publishers can enforce. In this way, ethics committees, institutions, and funders are arguably better placed to enforce prospective registration. For example, in the UK, the Health Research Authority has made trial registration a condition of receiving ethics approval [21].

Another limitation is the definition of a clinical trial. We applied the WHO definition of a clinical trial [7]. Initial ICMJE requirements for registration were “*any research project that prospectively assigns human subjects to intervention or comparison groups to study the cause-and-effect relationship between a medical intervention and a health outcome*”. This excludes those only examining pharmacokinetics or toxicity [6]. For all trials beginning participant enrolment from 1 July 2008 onwards, the ICMJE has used the WHO definition of a clinical trial [22]. We also identified manuscripts for inclusions using the word ‘trial’. It is possible that this could have led to missed studies.

Our sample size was too small to determine whether certain factors (such as competing interests or geographical location of authors) were associated with prospective versus retrospective registration.

## Conclusions

Despite the requirement for prospective registration of clinical trials in many journals for the past ten years, this study is the first to show that a large number of clinical trials were not prospectively registered.

The lack of prospective registration identified in this study demonstrates that more needs to be done to improve adherence to prospective registration. It also highlights the importance of allowing retrospective trial registration of studies that have not been prospectively registered in order to prevent the non-publication of potentially valuable research for which human participants have given up their time and exposed themselves to risk.

The results also suggest that there is a need for clear linkage of published articles reporting clinical trials to their trial registry records. This, along with the transparent inclusion of the date of registration in the published article and clear indication in the trial registry record when a trial has been registered retrospectively, will increase transparency and ensure that readers are aware of whether a clinical trial has been prospectively or retrospectively registered.

## Abbreviations

ANZCTR: Australian New Zealand Clinical Trials Registry
ChiCTR: Chinese Clinical Trial Register
DRKS: Deutsches Register Klinischer Studien (German Clinical Trials Register)
ICMJE: International Committee of Medical Journal Editors
NTR: Netherlands Trial Register
RCT: randomised controlled trial
TRN: trial registration number
UMIN-CTR: University Hospital Medical Information Network Clinical Trials Registry
